# Supplementary material for: End Sequence Analysis Toolkit (ESAT) expands the extractable information from single-cell RNA-seq data
Source: Genome Res. 2016 Oct;26(10):1397–410. doi: 10.1101/gr.207902.116 (PMC5052061; doi:10.1101/gr.207902.116)
Supplement: Supplemental Material [file supp_gr.207902.116_Supp_Materials.tar.gz › ESAT-esat_v0.1/doc/html/classumms_1_1esat_1_1_e_s_a_t.html]

ESAT: umms.esat.ESAT Class Reference


ESAT


- Main Page
- Related Pages
- Packages
- Classes
- Files

- Class List
- Class Index
- Class Hierarchy
- Class Members

All Classes Namespaces Files Functions Variables Enumerator Pages

- umms
- esat
- ESAT

Public Member Functions |
Static Public Member Functions |
Static Public Attributes |
List of all members

umms.esat.ESAT Class Reference

Collaboration diagram for umms.esat.ESAT:

[legend]

|  |  |
| --- | --- |
| Public Member Functions | |
|  | ESAT (String[] args) throws IOException, ParseException |
|  | |

|  |  |
| --- | --- |
| Static Public Member Functions | |
| static void | writeNormalizedMatrix (String fileName, MatrixWithHeaders resultMatrix) throws IOException |
|  | |
| static Map< String, Double > | calculateNormalizationFactors (MatrixWithHeaders mat) |
|  | |
| static List< Double > | array2List (double[] array) |
|  | |
| static Gene | getSubAnnotationFromEnd (Gene annotation, int length, int distanceFromTranscriptEnd) |
|  | |
| static Gene | getSubAnnotationFromStart (Gene annotation, int length, int distanceFromTranscriptStart) |
|  | |
| static BEDFileParser | reconstructGeneEnds (BEDFileParser completeReconstruction, BEDFileParser annotations, String annotationFile) throws IOException |
|  | |
| static void | main (String[] args) throws ParseException, IOException |
|  | |

|  |  |
| --- | --- |
| Static Public Attributes | |
| static String | whitespaceDelimiter = "\\s++" |
|  | |

## Detailed Description

Author
:   skadri

## Constructor & Destructor Documentation

|  |  |  |  |  |  |
| --- | --- | --- | --- | --- | --- |
| umms.esat.ESAT.ESAT | ( | String[] | *args* | ) | throws IOException, ParseException |

If user wants to reconstruct gene ends.

Here is the call graph for this function:

Here is the caller graph for this function:

## Member Function Documentation

|  |  |  |  |  |  |
| --- | --- | --- | --- | --- | --- |
| static List<Double> umms.esat.ESAT.array2List | ( | double[] | *array* | ) |  |

static

This function converts an array of double to List of type Double

Parameters
:   |  |  |
    | --- | --- |
    | double[] |  |

Returns

Here is the caller graph for this function:

|  |  |  |  |  |  |
| --- | --- | --- | --- | --- | --- |
| static Map<String,Double> umms.esat.ESAT.calculateNormalizationFactors | ( | MatrixWithHeaders | *mat* | ) |  |

static

Calculates the scaling factor for each column of the specified matrix, using the normalization method used by DESeq.

Parameters
:   |  |  |
    | --- | --- |
    | mat |  |

Returns

Here is the call graph for this function:

Here is the caller graph for this function:

|  |  |  |  |
| --- | --- | --- | --- |
| static Gene umms.esat.ESAT.getSubAnnotationFromEnd | ( | Gene | *annotation*, |
|  |  | int | *length*, |
|  |  | int | *distanceFromTranscriptEnd* |
|  | ) |  |  |

static

Returns the subannotation of length "length" at a distance "distanceFromTranscriptEnd" from the 3' end of the gene

Parameters
:   |  |  |
    | --- | --- |
    | annotation |  |
    | length |  |
    | distanceFromTranscriptEnd |  |

Returns

Here is the call graph for this function:

|  |  |  |  |
| --- | --- | --- | --- |
| static Gene umms.esat.ESAT.getSubAnnotationFromStart | ( | Gene | *annotation*, |
|  |  | int | *length*, |
|  |  | int | *distanceFromTranscriptStart* |
|  | ) |  |  |

static

Returns the subannotation of length "length" at a distance "distanceFromTranscriptStart" from the 5' end of the gene

Parameters
:   |  |  |
    | --- | --- |
    | annotation |  |
    | length |  |
    | distanceFromTranscriptEnd |  |

Returns

Here is the call graph for this function:

|  |  |  |  |  |  |
| --- | --- | --- | --- | --- | --- |
| static void umms.esat.ESAT.main | ( | String[] | *args* | ) | throws ParseException, IOException |

static

Parameters
:   |  |  |
    | --- | --- |
    | args |  |

Exceptions
:   |  |  |
    | --- | --- |
    | IOException |  |
    | ParseException |  |

Here is the call graph for this function:

|  |  |  |  |
| --- | --- | --- | --- |
| static BEDFileParser umms.esat.ESAT.reconstructGeneEnds | ( | BEDFileParser | *completeReconstruction*, |
|  |  | BEDFileParser | *annotations*, |
|  |  | String | *annotationFile* |
|  | ) |  | throws IOException |

static

Returns the reconstructed annotation set based on the reconstruction provided

Parameters
:   |  |  |
    | --- | --- |
    | completeReconstruction |  |
    | annotations |  |
    | annotationFile |  |

Returns

Exceptions
:   |  |  |
    | --- | --- |
    | IOException |  |

Here is the caller graph for this function:

|  |  |  |  |
| --- | --- | --- | --- |
| static void umms.esat.ESAT.writeNormalizedMatrix | ( | String | *fileName*, |
|  |  | MatrixWithHeaders | *resultMatrix* |
|  | ) |  | throws IOException |

static

This function takes an input count matrix and returns a normalized matrix, where a scaling factor is calculated for each sample, and all genes for that sample are scaled by this factor.

Parameters
:   |  |  |
    | --- | --- |
    | fileName |  |
    | resultMatrix |  |

Exceptions
:   |  |  |
    | --- | --- |
    | IOException |  |

Here is the call graph for this function:

Here is the caller graph for this function:

## Member Data Documentation

String umms.esat.ESAT.whitespaceDelimiter = "\\s++"

static

---

The documentation for this class was generated from the following file:

- java/umms/esat/ESAT.java


---

Generated on Fri Aug 15 2014 15:11:15 for ESAT by  

 1.8.7
